# Climbing the Dark Ladder: How Status and Inclusion Aspirations, Perceived Attainment, and Behaviors Relate to the Dark Triad

**DOI:** 10.3390/bs15091221

**Published:** 2025-09-08

**Authors:** Nikhila Mahadevan, Christian H. Jordan

**Affiliations:** 1 Department of Psychology, University of Essex, Colchester CO4 3SQ, UK; 2 Department of Psychology, Wilfrid Laurier University, Waterloo, ON N2L 3C5, Canada; cjordan@wlu.ca

**Keywords:** Dark Triad, grandiose narcissism, Machiavellianism, psychopathy, status, inclusion

## Abstract

Individual differences in the Dark Triad may partially reflect differences in interpersonal motivational patterns such as a strong desire for status. These studies examine how desires for status and inclusion, perceived attainment of status and inclusion, and status-seeking and inclusion-seeking behavior relate to the Dark Triad (grandiose narcissism, Machiavellianism, and psychopathy). Two studies (*N* = 591) find that individuals high in Dark Triad traits generally desire status, feel they have attained high status, and report behaving in status-seeking ways (once desires for inclusion, perceived attainment of inclusion, and inclusion-seeking behavior are controlled, respectively). They generally do not desire inclusion, do not feel they have attained inclusion, and do not report behaving in inclusion-seeking ways (once desires for status, perceived attainment of status, and status-seeking behavior are controlled, respectively). These associations are largely observed for the dimensions of the Dark Triad involving agentic extraversion and antagonism, but not for those involving impulsivity. This research delineates the motivational, social, and behavioral profile of the Dark Triad and its dimensions with implications for understanding the “core” of the Dark Triad.

## 1. Introduction

The Dark Triad refers to three socially undesirable personality traits—grandiose narcissism, Machiavellianism, and psychopathy—that vary normally in the general population (Paulhus, 2014; Paulhus & Williams, 2002). Do they share common social motivations that make them “dark” or socially aversive? This research investigates whether people high in these traits strongly desire status and weakly desire inclusion. Examining the interpersonal motives that characterize dark personalities can provide insight into the causes of their socially aversive behavior (Jonason & Ferrell, 2016; Jonason & Zeigler-Hill, 2018). To further delineate points of similarity and difference among the Dark Triad traits, we examine how they relate to perceptions of having achieved status and inclusion, and to status- and inclusion-relevant goal-related behavior. Finally, we examine how lower-order dimensions of these traits relate to these interpersonal outcomes. In doing so, we provide a more complete picture of the motivational, social, and behavioral profile of the Dark Triad in relation to both status and inclusion.

### 1.1. The Dark Triad

Grandiose narcissism, Machiavellianism, and psychopathy are positively correlated but distinct traits (Petrides et al., 2011). Grandiose narcissism is characterized by arrogance and superiority (Raskin & Terry, 1988; Rhodewalt & Morf, 1998). Grandiosely narcissistic individuals act authoritatively, feel entitled to special treatment and privileges, and lack empathy. Machiavellianism is characterized by cynicism and manipulation (Christie & Geis, 1970; Rauthmann & Will, 2011). Machiavellian individuals are emotionally cold, contemptuous of others, and willing to exploit them. Psychopathy is characterized by callousness and recklessness (Hare, 1985, 1991). Psychopathic individuals are thrill-seeking, violate social rules, and lack remorse (Hall & Benning, 2006).

Contemporary work on the Dark Triad indicates that each of the three traits is composed of multiple, lower-order dimensions. These include *narcissistic admiration* and *rivalry* (for grandiose narcissism; Back et al., 2013; Krizan & Herlache, 2018; Miller et al., 2017), *agency, antagonism,* and *planfulness* (for Machiavellianism; Collison et al., 2018; DeShong et al., 2017), and *boldness, meanness,* and *disinhibition* (for psychopathy; Patrick, 2010; Patrick et al., 2009). Notably, these lower-order dimensions share common variance. In terms of the five-factor model of personality, each Dark Triad trait has a dimension that reflects *agentic extraversion* (i.e., narcissistic admiration, agency, and boldness), and a dimension that reflects low agreeableness or *antagonism* (i.e., narcissistic rivalry, antagonism, and meanness). In addition, Machiavellianism and psychopathy each have a dimension that reflects high or low conscientiousness or *impulsivity*, respectively (i.e., planfulness and disinhibition).

### 1.2. Need for Social Status

Status refers to one’s position in a social hierarchy. It involves having others’ *respect* and *admiration* (Magee & Galinsky, 2008). Several lines of theory and research identify the desire for social status as being central to grandiose narcissism. *Hierometer theory* (Mahadevan et al., 2023a, 2023b) postulates that grandiose narcissism operates as a “hierometer”, which tracks people’s status in the social hierarchy and regulates status-seeking behavior. Higher status leads to higher narcissism and greater assertiveness, whereas lower status leads to lower narcissism and greater acquiescence (Mahadevan et al., 2016, 2020). The *extended agency model* (Campbell & Foster, 2007) postulates that the core characteristics of grandiose narcissism operate cohesively to facilitate status pursuit and attainment. Relatedly, several process models of narcissism postulate that narcissistic features function together to bolster an inflated self or high-status position (Back et al., 2013; Grapsas et al., 2020; Morf & Rhodewalt, 2001; Zeigler-Hill et al., 2019).

Consistent with such theorizing, grandiosely narcissistic individuals report an elevated desire for power and status (Carroll, 1987; Zeigler-Hill & Dehaghi, 2023). They are sensitive to fluctuations in their status (Mahadevan et al., 2019a; Zeigler-Hill et al., 2019), behave assertively in social situations (Mahadevan et al., 2016, 2020), and expect to achieve high status in future (Zitek & Jordan, 2016). They also prefer to pursue romantic relationships with high-status individuals (Campbell, 1999) and seek out leadership roles in groups (Grijalva et al., 2015). Thus, the desire for status may be a core motive underlying grandiose narcissism, explaining many of its features.

Comparatively little research has examined whether a desire for status also characterizes Machiavellianism and psychopathy. If it does, this desire may be a shared motive that helps explain these traits’ relations to socially aversive behaviors. Research on the similarities, or “core,” of the Dark Triad has tended to focus on examining their shared structural correlates or the nature of their shared variance. This research suggests that the core of the Dark Triad may reflect callousness and manipulation (Jones & Figueredo, 2013), antagonism or disagreeableness (Vize et al., 2020), low honesty-humility (Book et al., 2015; Lee & Ashton, 2014), or a general dark (D) factor (Moshagen et al., 2018; Schreiber & Marcus, 2020). In contrast, some research highlights the importance of considering common motivational patterns that characterize dark traits (Jonason & Ferrell, 2016; Jonason & Zeigler-Hill, 2018). In this view, individual differences in the Dark Triad may partially reflect differences in functional motivational patterns. We adopt this perspective to better understand the interpersonal motives that characterize individuals high in Dark Triad traits. Specifically, we investigate whether a desire for status characterizes all Dark Triad traits, particularly when a desire for inclusion is controlled.

If a desire for status characterizes all Dark Triad traits, it is important to further specify which aspects of these traits account for this motivation. Understanding which lower-order dimensions relate to a desire for status can delineate the common and unique motivations underlying each Dark Triad trait. This is important for understanding which dimensions may contribute to their “darkness.”

Bader et al. (2022) contend that the shared variance in dark personalities, or the D factor, overlaps mainly with antagonistic features (i.e., rivalry, antagonism, and meanness), which seems consistent with findings that a higher-order D factor also converges with the honesty-humility factor of the HEXACO model (Book et al., 2015; Schreiber & Marcus, 2020). In these views, other dimensions give dark personalities unique characteristics but may contribute relatively little to socially aversive behavior and would not be expected to relate consistently to an overriding desire for status, particularly at the expense of inclusion. Other perspectives suggest that the features of Dark Triad traits cohere because they reflect a “coordinated system” that confers advantages to individuals high in these traits in certain environments (Book et al., 2015; Jonason et al., 2010). In this view, all or most Dark Triad dimensions will relate to similar social motives because they work in a coordinated fashion to help achieve social goals. It is therefore theoretically important to understand which Dark Triad dimensions are characterized by a strong desire for status.

### 1.3. Status and Inclusion: Aspirations, Perceived Attainment, and Behavior

To gain a more complete picture of the motivational, social, and behavioral profile of the Dark Triad, we additionally investigate how these traits relate to desire for inclusion, perceived attainment of status and inclusion, and status-seeking and inclusion-seeking behavior. Whereas status reflects a person’s position in the social hierarchy and involves being respected and admired by others, inclusion reflects a person’s position in the social collective and involves being liked and accepted by others (Gregg et al., 2017a, 2017b). Status involves standing out, whereas inclusion involves fitting in (Mahadevan et al., 2021).

Contemporary research on motivation distinguishes between the need for status and need for inclusion (also known as the need to belong; Anderson et al., 2015; Baumeister & Leary, 1995). Both motives are considered fundamental—distinct and non-redundant (Anderson et al., 2015). However, despite being fundamental, people nevertheless differ in the degree to which they experience them (Sheldon & Schüler, 2011). Some people have high status aspirations (i.e., strongly crave respect and admiration), whereas others have low status aspirations (i.e., do not strongly crave respect and admiration). Likewise, some people have high inclusion aspirations (i.e., strongly crave liking and acceptance), whereas others have low inclusion aspirations (i.e., do not strongly crave liking and acceptance). We investigate how individual differences in aspirations for status and inclusion relate to the Dark Triad.

People also differ in the degree to which they have attained—or see themselves as having attained—status and inclusion (Fournier, 2009; Mahadevan, 2024). Whereas some people have high status attainment (i.e., are highly respected and admired), others have low status attainment (i.e., are not very highly respected and admired). Likewise, whereas some people have high inclusion attainment (i.e., are well-liked and accepted), others have low inclusion attainment (i.e., are not very well-liked and accepted). We additionally investigate how individual differences in perceived attainment of status and inclusion relate to the Dark Triad.

Finally, people differ in their status-seeking and inclusion-seeking behavior (Markey et al., 2015; Wiggins, 1991). We use the term ‘assertiveness’ to refer to behavior which falls along the dominance–submission axis of the agency–communion circumplex (Mahadevan et al., 2016). Behaving assertively involves taking charge, assigning others to tasks, and being forthright about one’s opinions and decisions. It reflects and promotes status attainment (Mahadevan et al., 2020). We use the term ‘affiliativeness’ to refer behavior which falls along the agreeableness–quarrelsomeness axis of the agency–communion circumplex (Mahadevan et al., 2016). Behaving affiliatively involves being friendly, expressing affection and reassurance, and helping others. It reflects and promotes inclusion attainment (Mahadevan et al., 2020). We additionally investigate how individual differences in perceived assertiveness and affiliativeness relate to the Dark Triad.

### 1.4. Values, Motivations and Behaviors Associated with the Dark Triad

Individuals high in Dark Triad traits have a value system that prioritizes agency over communion. All three traits are located in Quadrant-II of the interpersonal circumplex (Wiggins, 1979, 1996), associated with high agency and low communion (Jones & Paulhus, 2010). They aspire for fame (Southard & Zeigler-Hill, 2016), money (Lee et al., 2013), leadership (Guillén et al., 2023), and social dominance (Jones & Figueredo, 2013). In terms of Schwartz’s (1992) universal social values model, Dark Triad traits correlate positively with achievement, power, hedonism, and stimulation and negatively with benevolence, universalism, self-direction, and tradition (Jonason et al., 2020a; Kajonius et al., 2015). In terms of Trapnell and Paulhus’ (2012) agentic and communal values model, they correlate positively with ambition, power, and influence and negatively with altruism, compassion, and belonging. Overall, people high in Dark Triad traits possess self-enhancing or agentic values rather than self-transcending or communal values (Jonason et al., 2015). Dark Triad traits may reflect a pattern of prioritizing agency over communion as a coping response to early experiences of ostracism (Pu & Gan, 2025).

People high in Dark Triad traits also desire dominance (Semenyna & Honey, 2015). They report enhanced desires for power, hierarchical position, and leadership (Jonason & Ferrell, 2016; Jonason & Zeigler-Hill, 2018; Lee et al., 2013). People also score higher on Dark Triad traits, especially grandiose narcissism, in countries that emphasize hierarchy, competitiveness, and status differences (Jonason et al., 2020b).

Finally, people high in Dark Triad traits behave in selfish and antisocial ways. All three traits predict unethical workplace behaviors including theft, fraud, sabotage, and abusive supervision (Harrison et al., 2018; O’Boyle et al., 2012). They are also associated with physical and verbal aggression, cyber-bullying, and cyber-trolling (Goodboy & Martin, 2015; Kjærvik & Bushman, 2021; Leite et al., 2023). Finally, all three traits predict infidelity, lack of commitment, and sexual coercion in romantic relationships (Brewer et al., 2015; Figueredo et al., 2015; Jonason et al., 2012).

### 1.5. Contribution of the Current Research

In sum, prior research indicates that individuals high in Dark Triad traits possess self-enhancing values, desire power and dominance, and behave in callous and self-centered ways. The current research replicates and extends these findings in order to test whether a strong desire for status (relative to inclusion) is a shared social motivation, which underlies their socially aversive tendencies. It does so in several ways.

First, whereas prior studies have examined motives for power, dominance, or hierarchical position, the current research examines how Dark Triad traits relate to desire for status in the form of social respect and admiration, specifically. Anderson et al. (2015) argue that status is a fundamental motive distinct from power and dominance. Whereas power concerns the control of resources, and dominance the ability to influence others through coercion, status involves voluntary deference rooted in respect and admiration. This form of status is sometimes called prestige because it is rooted in social perceptions and evaluations (Magee & Galinsky, 2008). In the current research, we focus specifically on the desire for status, in terms of striving to be respected and admired, and contrast it with the desire for inclusion, in terms of striving to be liked and accepted. We test whether each Dark Triad trait is associated with the desire for status after controlling desire for inclusion, and vice versa.

Second, whereas prior studies have examined specific interpersonal contexts, such as workplace relationships and romantic relationships, the current research examines how Dark Triad traits relate to perceived status attainment and perceived inclusion attainment, more generally. It tests whether each Dark Triad trait is associated with perceived status attainment after controlling perceived inclusion attainment, and vice versa.

Third, whereas prior studies have examined selfish, unethical, and aggressive behaviors, the current research examines how Dark Triad traits relate to status-seeking and inclusion-seeking behaviors specifically, in the form of interpersonal assertiveness and affiliativeness. It tests whether each Dark Triad trait is associated with interpersonal assertiveness after controlling interpersonal affiliativeness, and vice versa.

Finally, going beyond prior studies, to gain a more complete picture of the motivational, social, and behavioral profile of the Dark Triad, the current research examines how lower-order Dark Triad dimensions relate to desires for status and inclusion, perceived status and inclusion attainment, and status-seeking and inclusion-seeking behavior.

### 1.6. Hypotheses

We hypothesized that all Dark Triad traits would correlate positively with status aspirations (once inclusion aspirations were controlled) and negatively with inclusion aspirations (once status aspirations were controlled). We hypothesized that all Dark Triad traits would correlate positively with assertiveness (once affiliativeness was controlled) and negatively with affiliativeness (once assertiveness was controlled). Because grandiose narcissism relates to viewing oneself as having successfully attained status (Mahadevan & Jordan, 2022), we hypothesized similar results for Machiavellianism and psychopathy, given the shared variance across the lower-order Dark Triad dimensions. Because of their overlap with conscientiousness, which strongly predicts goal achievement (Wilmot & Ones, 2019), we expected the planfulness of those high in Machiavellianism to facilitate status attainment and the disinhibition of those high in psychopathy to impede it (Persson & Lilienfeld, 2019).

### 1.7. Overview

We conducted two studies. Study 1 examined the links among the Dark Triad, aspirations for status and inclusion, and perceived attainment of status and inclusion using traditional measures of grandiose narcissism, Machiavellianism and psychopathy, which provide an overall assessment of each trait. Study 2 examined the links among the Dark Triad, aspirations for status and inclusion, perceived attainment of status and inclusion, and assertiveness and affiliativeness using newer measures of grandiose narcissism, Machiavellianism and psychopathy, which assess lower-order dimensions of each trait.

In both studies, we first examined the zero-order correlations to understand the overall relations among each Dark Triad trait and each social outcome. Next, we tested unique relations of each Dark Triad trait with each status-related variable controlling the corresponding inclusion-related variable (and vice versa): We examined the links among each Dark Triad trait, status aspirations, perceived status attainment, and assertiveness controlling for inclusion aspirations, perceived inclusion attainment, and affiliativeness, respectively (and vice versa). Status- and inclusion-related constructs often correlate positively, perhaps reflecting a common motive for social validation, or the belief that achieving one helps to achieve the other (e.g., Fournier, 2009). Examining the unique associations of each status-related and inclusion-related variable with each Dark Triad trait provides a clearer sense of how they relate to unique status-related or inclusion-related motivations, social positions, and behaviors.

All subjects gave their informed consent for inclusion before they participated in the studies. The studies were conducted in accordance with the Declaration of Helsinki, and the protocol was approved by the Ethics Committee of the University of Essex (protocol codes: Study 1: ETH2021-1857; Study 2: ETH2122-0064).

In both studies, we aimed for sample sizes of at least 250. This sample size permits detection of small-to-medium effects of ρ ≥ 0.20 with 90% power at (two-tailed) α = 0.05 (the average effect size in social and personality psychology; Richard et al., 2003). In addition, correlation and regression estimates stabilize at this sample size (Schönbrodt & Perugini, 2013). However, we deliberately oversampled in anticipation of data exclusions. Post hoc sensitivity analyses indicate that Study 1’s final sample of 281 permits detection of small-to-medium effects of ρ ≥ 0.19, and Study 2’s final sample of 310 permits detection of small-to-medium effects of ρ ≥ 0.18, with 90% power at (two-tailed) α = 0.05. We did not stop and restart data collection at any point. We analyzed the data only once the full sample had been collected. The data and analysis codes are available at: https://osf.io/79rfc/?view_only=c877b835480d4301a44f08f37f0e8ab6 (deposited on 1 April 2025).

## 2. Study 1

Study 1 examined how aspirations for, and perceived attainment of, status and inclusion relate to the Dark Triad traits. We used the measures most frequently used to assess these traits, namely, the Narcissistic Personality Inventory, the Mach-IV Scale, and the Self-Report Psychopathy Scale (Furnham et al., 2013), which provide an overall assessment of each trait. Accordingly, Study 1 examined how overall levels of grandiose narcissism, Machiavellianism, and psychopathy relate to aspirations for, and perceived attainment of, status and inclusion.

### 2.1. Method

#### 2.1.1. Procedure

We hosted the study online on Qualtrics^TM^ and recruited participants via the leading research recruitment platform, Prolific.ac^TM^. Participants clicked on the link to the study and read information about the study’s aims and objectives. They then indicated their consent to taking part by checking a box and completed the study questionnaires and demographic information.

#### 2.1.2. Participants

The study sample was drawn from the general population. Any adult (aged 18 or older) who was resident in the U.S.A. and fluent in English was eligible to take part. No other pre-screening criteria were used.

A total of 302 participants completed the study. Although online research recruitment platforms such as Prolific.ac^TM^ generally yield high-quality data (Germine et al., 2012), it is nonetheless prudent to check data quality and exclude participants whose data, for any of several reasons, appear to be suspicious and/or of poor quality. Accordingly, consistent with best practices in the field and with prior work (Gregg et al., 2018), we excluded 21 participants for one or more of the following reasons: (a) reported being aged below 18 (i.e., were below the age of consent; 0.3%); (b) reported poor English proficiency (i.e., might be unable to fully comprehend the questions; 0.0%); (c) participated multiple times (making it impossible to ascertain which set of responses, if any, were genuine, 2.0%); (d) completed the study very quickly (in less than one-third of the median completion time), suggesting an absence of thoughtful responding (1.7%); (e) had too much missing data (over 10% of questionnaire items), suggesting careless responding (0.0%); (f) showed unvarying responses to questionnaires with both forward-coded and reverse-coded items, suggesting not having read or understood the questions properly (3.3%); (g) reported their data were not trustworthy (0.0%); or (h) were multivariate outliers (0.7%).

The final (community) sample consisted of 281 participants (142 men, 133 women, 4 other, and 2 who did not specify their gender), aged 18–73 years (*M* = 33.03, *SD* = 12.05). They reported their English language proficiency as being excellent (86.7%), good (12.5%), or average (0.7%). They identified their ethnicity as White/European (62.7%), Southeast Asian (16.1%), Black/African/Caribbean (11.1%), Latin American (3.9%), South Asian (1.8%), Aboriginals/First Nations/Metis (0.4%), West Asian (0.4%), or other (3.6%). They identified their country of origin as the United States of America (85.5%), China (2.5%), Malaysia (1.1%), Philippines (1.1%), Germany (0.7%), Japan (0.7%), Mexico (0.7%), Australia (0.4%), Bulgaria (0.4%), Canada (0.4%), Cuba (0.4%), Eritrea (0.4%), Ethiopia (0.4%), Guatemala (0.4%), Hong Kong (0.4%), India (0.4%), Italy (0.4%), Jamaica (0.4%), the Netherlands (0.4%), Poland (0.4%), Republic of Korea (0.4%), South Korea (0.4%), Thailand (0.4%), Ukraine (0.4%), United Kingdom (0.4%), Uzbekistan (0.4%), Venezuela (0.4%), or Vietnam (0.4%).

### 2.2. Measures

#### 2.2.1. Status Aspirations

We assessed status aspirations using a 10-item questionnaire (Mahadevan et al., 2019b). Participants indicated the extent to which they desire status (e.g., “Having the respect of others is essential to me”; 1 = *strongly disagree*, 5 = *strongly agree*).

#### 2.2.2. Inclusion Aspirations

We assessed inclusion aspirations using a parallel 10-item questionnaire (Mahadevan et al., 2019b). Participants indicated the extent to which they desire inclusion (e.g., “Being liked by others is essential to me”; 1 = *strongly disagree*, 5 = *strongly agree*).

#### 2.2.3. Perceived Status Attainment

We assessed perceived status attainment using an 8-item questionnaire (Huo et al., 2010; Mahadevan et al., 2016). Participants indicated the extent to which they feel that others respect and admire them (e.g., “Most of the time I feel that people admire me”; 1 = *strongly disagree*, 5 = *strongly agree*).

#### 2.2.4. Perceived Inclusion Attainment

We assessed perceived inclusion attainment using a parallel 9-item questionnaire (Huo et al., 2010; Mahadevan et al., 2016). Participants indicated the extent to which they feel that others like and accept them (e.g., “Most of the time I feel that people accept me”; 1 = *strongly disagree*, 5 = *strongly agree*).

#### 2.2.5. Grandiose Narcissism

We assessed grandiose narcissism using the 40-item *Narcissistic Personality Inventory* (NPI; Raskin & Terry, 1988). It consists of 40 pairs of statements. Each contains one narcissistic statement (e.g., “I really like to be the center of attention”) and one non-narcissistic statement (e.g., “It makes me uncomfortable to be the center of attention”). Participants chose the statement that fit them better.

#### 2.2.6. Machiavellianism

We assessed Machiavellianism using the 20-item *Mach-IV Scale* (Christie & Geis, 1970). Participants indicated their agreement with statements that reflect a general tendency to be Machiavellian (e.g., “It is wise to flatter important people”; 1 = *strongly disagree*, 5 = *strongly agree*).^1^

#### 2.2.7. Psychopathy

We assessed psychopathy using the 34-item *Self-Report Psychopathy Scale* (SRP-III; Williams & Paulhus, 2004). Participants indicated their agreement with statements that reflect a general tendency toward psychopathy (e.g., “I have broken into a building or vehicle in order to steal something or to vandalize”; 1 = *strongly disagree*, 5 = *strongly agree*).

### 2.3. Results

We report descriptive statistics, internal consistencies, and zero-order correlations in Table 1. All measures showed good internal consistency. Status aspirations correlated positively with inclusion aspirations. Perceived status attainment correlated positively with perceived inclusion attainment. Thus, participants who desired higher status also desired to be more included, and participants who felt they had attained higher status also felt they were more included.

#### 2.3.1. Zero-Order Correlations

Status aspirations correlated positively with all Dark Triad traits. Inclusion aspirations correlated positively with grandiose narcissism and psychopathy, and marginally positively with Machiavellianism.^2^ Perceived status attainment correlated positively with grandiose narcissism, was unrelated to psychopathy, and correlated negatively with Machiavellianism. Perceived inclusion attainment correlated positively with grandiose narcissism and negatively with Machiavellianism and psychopathy.

#### 2.3.2. Regression Analyses

To examine unique associations, we regressed each Dark Triad measure (individually) onto status aspirations and inclusion aspirations (simultaneously), and onto perceived status attainment and perceived inclusion attainment (simultaneously). We report the standardized regression coefficients in Table 2.

Status aspirations remained positively related to grandiose narcissism, *t*(278) = 8.02, *p* < 0.001, Machiavellianism, *t*(278) = 2.70, *p* = 0.007, and psychopathy, *t*(278) = 4.10, *p* < 0.001. In contrast, inclusion aspirations no longer related positively to grandiose narcissism, *t*(278) = 0.72, *p* = 0.943, Machiavellianism, *t*(278) = −0.16, *p* = 0.870, and psychopathy, *t*(278) = 0.23, *p* = 0.819. This suggests the zero-order associations between the Dark Triad and inclusion aspirations reflect shared variance with status aspirations: Those high in Dark Triad traits may desire inclusion primarily as a means to enhance status.

Perceived status attainment remained positively related to grandiose narcissism, *t*(278) = 6.86, *p* < 0.001, became positively related to psychopathy, *t*(278) = 4.66, *p* < 0.001, and went from negatively related to unrelated to Machiavellianism, *t*(278) = 0.86, *p* = 0.389. In contrast, perceived inclusion attainment no longer related positively to grandiose narcissism, *t*(278) = −1.57, *p* = 0.117, remained negatively related to Machiavellianism, *t*(278) = −4.23, *p* < 0.001, and became more negatively related to psychopathy, *t*(278) = −4.93, *p* < 0.001.^3^

### 2.4. Discussion

All Dark Triad traits were positively associated with status aspirations. Likewise, they were positively associated with perceived status attainment (when Machiavellianism was assessed by the Trimmed-Mach but not the Mach-IV) once perceived inclusion attainment was controlled. This suggests that individuals high in Dark Triad traits may desire high status and feel they have achieved it, once the tendency to feel they are not well-liked is controlled. In contrast, once status aspirations were controlled, Dark Triad traits were unrelated to inclusion aspirations, and once perceived status attainment was controlled, were negatively related or unrelated to perceived inclusion attainment. Notably, when the shared variance between perceived status attainment and inclusion attainment was controlled, the relation of each Dark Triad trait with perceived status attainment became more positive, and with perceived inclusion attainment became more negative, sometimes substantially. This shared variance may reflect a sense of being generally perceived positively by others. These results suggest that individuals with dark personalities may not strongly strive to be included by others, and may not feel they are especially included, once a general sense of being socially validated is controlled. They may seek inclusion primarily as a means to status rather than for its own sake.

## 3. Study 2

Study 1 suggests that Dark Triad traits are characterized by a common motivation to gain respect and admiration. Study 2 had three main goals. First, it assessed the Dark Triad with different measures to see if the results of Study 1 replicate. Second, in addition to aspirations for, and perceived attainment of, status and inclusion, it examined interpersonal behavior: specifically, how assertiveness and affiliativeness relate to the Dark Triad. Third, it examined the lower-order dimensions of the Dark Triad. We assessed grandiose narcissism, Machiavellianism, and psychopathy with the Narcissistic Admiration and Rivalry Questionnaire (NARQ), the Five Factor Machiavellianism Inventory (FFMI), and the Triarchic Psychopathy Measure (TPM), respectively, to test how lower-order Dark Triad dimensions relate to aspirations for status and inclusion, perceived attainment of status and inclusion, and assertiveness and affiliativeness.

Each Dark Triad trait consists of multiple, lower-order dimensions. The Narcissistic Admiration and Rivalry Concept model of grandiose narcissism (Back et al., 2013) delineates two dimensions—narcissistic admiration and rivalry—which reflect assertive self-enhancement and antagonistic self-protection, respectively (Krizan & Herlache, 2018; Miller et al., 2017). The Five Factor model of Machiavellianism delineates three dimensions—agency, antagonism, and planfulness—which mirror the Big Five traits of extraversion, agreeableness, and conscientiousness, respectively (Collison et al., 2018). Agency reflects confidence and assuredness, antagonism reflects cynicism and duplicity, and planfulness reflects deliberation and self-control (DeShong et al., 2017). Similarly, the triarchic model of psychopathy delineates three dimensions—boldness, meanness, and disinhibition—where boldness reflects venturesomeness and fearlessness, meanness reflects callousness and aggression, and disinhibition reflects impulsivity (Patrick et al., 2009).

Notably, whereas Machiavellianism is characterized by planfulness (i.e., low impulsivity), psychopathy is characterized by disinhibition (i.e., high impulsivity; Jones & Paulhus, 2011). However, traditional measures of Machiavellianism and psychopathy, such as the Mach-IV and the SRP-III (used in Study 1), arguably do not distinguish between the two well (Collison et al., 2018; DeShong et al., 2017). Accordingly, in Study 2, we used the FFMI and the TPM to assess Machiavellianism and psychopathy, respectively.

Importantly, these lower-order Dark Triad dimensions overlap. Narcissistic admiration, agency, and boldness involve agentic extraversion; narcissistic rivalry, antagonism, and meanness involve disagreeableness or antagonism; and planfulness and disinhibition involve conscientiousness or impulsivity (at opposite poles). Failure to consider component dimensions of Dark Triad traits can hinder efforts to identify where, and how, these constructs converge and diverge (Rose et al., 2023). In Study 1, the Dark Triad traits differed somewhat in how they related to status attainment and inclusion attainment; it is unclear how much these differences reflect how their underlying dimensions are represented in the unidimensional measures used in Study 1. Accordingly, in Study 2, we used measures that cover these dimensions and conducted dimension-specific analyses of how the Dark Triad traits relate to aspirations for status and inclusion, perceived attainment of status and inclusion, assertiveness and affiliativeness.

### 3.1. Method

#### 3.1.1. Procedure

Study 2 followed the same method and procedure as Study 1. It was hosted online on Qualtrics^TM^, and participants were recruited via Prolific.ac^TM^.

#### 3.1.2. Participants

As in Study 1, the sample was drawn from the general population. It was open to adult residents of the U.S.A. (aged 18 and above) who were fluent in English. No other pre-screening criteria were used.

A total of 326 participants completed the study. We again scrutinized the data carefully and excluded 16 participants whose data appeared to be suspicious and/or of poor quality, for the same reasons as in Study 1 (underage: 0.0%; poor English proficiency: 0.0%; multiple completions: 2.5%; overly rapid completion: 0.3%; missing data: 0.0%; unvarying responses: 1.5%; untrustworthy data: 0.0%; or multivariate outliers: 0.6%).

The final (community) sample consisted of 310 participants (127 men, 180 women, and 1 other), aged 18–82 years (*M* = 30.85, *SD* = 11.33). They reported their English language proficiency as being excellent (91.9%), good (7.1%), or average (0.9%). They identified their ethnicity as White/European (61.3%), Black/African/Caribbean (15.2%), Latin American (11.3%), Southeast Asian (6.8%), South Asian (1.9%), West Asian (1.0%), Arab (0.6%), or other (1.9%). They identified their country of origin as the United States of America (80.8%), Mexico (1.7%), Philippines (1.3%), Brazil (1.3%), Venezuela (1.0%), Cuba (0.7%), Dominican Republic (0.7%), El Salvador (0.7%), Germany (0.7%), India (0.7%), Jamaica (0.7%), Japan (0.7%), Russian Federation (0.7%), South Korea (0.7%), United Kingdom (0.7%), Argentina (0.3%), Bahamas (0.3%), Bangladesh (0.3%), Belgium (0.3%), China (0.3%), France (0.3%), Ghana (0.3%), Honduras (0.3%), Hong Kong (0.3%), Israel (0.3%), Italy (0.3%), Kenya (0.3%), Malaysia (0.3%), Nepal (0.3%), Nigeria (0.3%), Portugal (0.3%), Serbia (0.3%), South Africa (0.3%), Thailand (0.3%), Tunisia (0.3%), or Ukraine (0.3%).

### 3.2. Measures

We assessed status aspirations, inclusion aspirations, perceived status attainment, and perceived inclusion attainment with the same measures as in Study 1.

#### 3.2.1. Interpersonal Behavior

We assessed assertive and affiliative behavior using the 48-item *Social Behavior Inventory* (SBI; Moskowitz, 1994). It consists of four 12-item sub-scales that assess dominant (e.g., “I assign others to tasks”), submissive (e.g., “I let others make plans or decisions”), agreeable (e.g., “I smile and laugh with others”), and quarrelsome (e.g., “I make sarcastic comments”) behavior (1 = *very unlike me*, 6 = *very like me*). Consistent with past research, we averaged items across the dominant and submissive sub-scales to create an assertiveness score and averaged items across the agreeable and quarrelsome sub-scales to create an affiliativeness score (Mahadevan et al., 2016, 2020).

#### 3.2.2. Grandiose Narcissism

We assessed grandiose narcissism using the 18-item *Narcissistic Admiration and Rivalry Questionnaire* (Back et al., 2013). It assesses two dimensions: narcissistic admiration (e.g., “I show others how special I am”) and narcissistic rivalry (e.g., “I enjoy it when another person is inferior to me”; 1 = *not agree at all*, 6 = *agree completely*). We averaged all items to create an overall grandiose narcissism score and sub-scale items to create narcissistic admiration and rivalry scores.

#### 3.2.3. Machiavellianism

We assessed Machiavellianism using the 52-item *Five Factor Machiavellianism Inventory* (Collison et al., 2018). It assesses three dimensions: agency (e.g., “People look to me to get the job done”), antagonism (e.g., “I don’t worry about other people’s needs if they conflict with my own”), and planfulness (e.g., “I like to carefully consider the consequences before I make a decision”; 1 = *strongly disagree*, 5 = *strongly agree*). We averaged all items to create an overall Machiavellianism score and sub-scale items to create agency, antagonism, and planfulness scores.

#### 3.2.4. Psychopathy

We assessed psychopathy using the 58-item *Triarchic Psychopathy Measure* (Patrick, 2010). It assesses three dimensions: boldness (e.g., “I’m afraid of far fewer things than most people”), meanness (e.g., “It doesn’t bother me to see someone else in pain”), and disinhibition (e.g., “I get in trouble for not considering the consequences of my actions; 1 = *false*, 2 = *somewhat false*, 3 = *somewhat true*, 4 = *true*). We averaged all items to create an overall psychopathy score and sub-scale items to create boldness, meanness and disinhibition scores.

### 3.3. Results

We report descriptive statistics, internal consistencies, and zero-order correlations in Table 3. All measures and sub-scales showed good internal consistency. As in Study 1, status aspirations correlated positively with inclusion aspirations, and perceived status attainment correlated positively with perceived inclusion attainment. Assertiveness and affiliativeness were uncorrelated.

#### 3.3.1. Zero-Order Correlations

Status aspirations correlated positively with grandiose narcissism, Machiavellianism, and psychopathy (Table 3). Inclusion aspirations correlated positively with grandiose narcissism, marginally positively with psychopathy, and were unrelated to Machiavellianism. Perceived status attainment correlated positively with all Dark Triad traits. Perceived inclusion attainment correlated positively with grandiose narcissism and Machiavellianism and was unrelated to psychopathy. Assertiveness correlated positively with all Dark Triad traits. Affiliativeness correlated negatively with grandiose narcissism and Machiavellianism, and marginally negatively with psychopathy.

#### 3.3.2. Regression Analyses

We examined unique associations by regressing each Dark Triad measure (individually) onto status aspirations and inclusion aspirations (simultaneously), onto perceived status attainment and perceived inclusion attainment (simultaneously), and onto assertiveness and affiliativeness (simultaneously). We report the standardized regression coefficients in Table 2.

Status aspirations remained positively related to narcissism, *t*(307) = 8.85, *p* < 0.001, Machiavellianism, *t*(307) = 9.50, *p* < 0.001, and psychopathy, *t*(307) = 5.10, *p* < 0.001. In contrast, inclusion aspirations became unrelated to narcissism, *t*(307) = 1.61, *p* = 0.107, remained unrelated to psychopathy, *t*(307) = −1.15, *p* = 0.252, and became negatively related to Machiavellianism, *t*(307) = −5.82, *p* < 0.001. This suggests the zero-order associations between the Dark Triad traits and inclusion aspirations reflect shared variance with status aspirations; individuals high in Dark Triad traits may not particularly desire inclusion and may even prefer to avoid it (in the case of Machiavellianism), once status aspirations are controlled. To the extent that those high in narcissism and psychopathy want to be liked and accepted, it may reflect a general desire for social validation or striving to be respected and admired.

Perceived status attainment remained positively related to grandiose narcissism, *t*(307) = 8.17, *p* < 0.001, Machiavellianism, *t*(307) = 9.46, *p* < 0.001, and psychopathy, *t*(307) = 6.25, *p* < 0.001. In contrast, perceived inclusion attainment became negatively related to narcissism, *t*(307) = −2.74, *p* < 0.001, and psychopathy, *t*(307) = −4.25, β = −0.32, *p* < 0.001, and unrelated to Machiavellianism, *t*(307) = −0.32, *p* = 0.753. This suggests the zero-order associations between the Dark Triad and perceived inclusion attainment may reflect shared variance with status attainment: Individuals high in Dark Triad constructs may not feel particularly included, once perceived status attainment is controlled.

Assertiveness remained positively related to grandiose narcissism, *t*(307) = 6.94, *p* < 0.001, Machiavellianism, *t*(307) = 15.16, *p* < 0.001, and psychopathy, *t*(307) = 8.18, *p* < 0.001. In contrast, affiliativeness remained negatively related to grandiose narcissism, *t*(307) = −3.72, *p* < 0.001, Machiavellianism, *t*(307) = −2.41, *p* = 0.017, and psychopathy, *t*(306) = −9.43, *p* < 0.001. This suggests individuals high in Dark Triad traits adopt a relatively assertive, but disagreeable, behavioral style towards others in interpersonal situations.

#### 3.3.3. Zero-Order Correlations with Dark Triad Dimensions

Status aspirations correlated positively with all Dark Triad dimensions, except planfulness. Inclusion aspirations correlated positively with narcissistic admiration, narcissistic rivalry, and disinhibition, but no other dimensions (Table 4).

Perceived status attainment correlated positively with dimensions associated with agentic extraversion (narcissistic admiration, agency and boldness), and planfulness. It was uncorrelated with dimensions associated with antagonism (narcissistic rivalry, antagonism, and meanness), and disinhibition. Perceived inclusion attainment correlated positively with dimensions associated with agentic extraversion (narcissistic admiration, agency, and boldness), and planfulness, but negatively with dimensions associated with antagonism (narcissistic rivalry, antagonism, and meanness), and disinhibition.

Assertiveness correlated positively with dimensions associated with agentic extraversion (narcissistic admiration, agency, and boldness) and Machiavellian antagonism. It was uncorrelated with narcissistic rivalry, meanness, planfulness, and disinhibition. Affiliativeness correlated positively with agency, boldness, and planfulness, but was uncorrelated with narcissistic admiration. It correlated negatively with dimensions associated with antagonism (narcissistic rivalry, antagonism, and meanness), and disinhibition.

#### 3.3.4. Regression Analyses with the Dark Triad Dimensions

To test unique associations with Dark Triad dimensions, we regressed each dimension (individually) onto status aspirations and inclusion aspirations (simultaneously), onto perceived status attainment and perceived inclusion attainment (simultaneously), and onto assertiveness and affiliativeness (simultaneously).

Status aspirations remained positively related to dimensions associated with agentic extraversion: narcissistic admiration, *t*(307) = 9.81, *p* < 0.001, agency, *t*(307) = 8.17, *p* < 0.001, and boldness, *t*(307) = 5.53, *p* < 0.001; and dimensions related to antagonism: narcissistic rivalry, *t*(307) = 3.10, *p* = 0.002, Machiavellian antagonism, *t*(307) = 4.60, *p* < 0.001, and meanness, *t*(307) = 2.71, *p* = 0.007; but were unrelated to planfulness, *t*(307) = 0.30, *p* = 0.764, and disinhibition, *t*(307) = 1.48, β = 0.10, *p* = 0.140. Inclusion aspirations became unrelated, or more negatively related, to dimensions associated with agentic extraversion: narcissistic admiration, *t*(307) = −0.21, *p* = 0.838, agency, *t*(307) = −5.51, *p* < 0.001, and boldness, *t*(307) = −3.63, *p* < 0.001. With the exception of narcissistic rivalry, inclusion aspirations were also unrelated to dimensions associated with antagonism: narcissistic rivalry, *t*(307) = 2.86, *p* = 0.005, Machiavellian antagonism, *t*(307) = −1.54, *p* = 0.125, and meanness, *t*(307) = −0.112, *p* = 0.911. Inclusion aspirations remained positively related to disinhibition, *t*(307) = 2.16, *p* = 0.031, and unrelated to planfulness, *t*(307) = −1.19, *p* = 0.236.

Perceived status attainment remained positively related to dimensions associated with agentic extraversion: narcissistic admiration, *t*(307) = 10.78, β = 0.65, *p* < 0.001, agency, *t*(307) = 9.43, β = 0.54, *p* < 0.001, and boldness, *t*(307) = 7.97, β = 0.51, *p* < 0.001. It became positively associated with all dimensions related to antagonism: narcissistic rivalry, *t*(307) = 2.76, β = 0.21, *p* = 0.006, Machiavellian antagonism, *t*(307) = 4.43, β = 0.33, *p* < 0.001, and meanness, *t*(307) = 4.72, *p* < 0.001. It became unrelated to planfulness, *t*(307) = −0.06, *p* = 0.954 and disinhibition, *t*(307) = 0.59, β = 0.05, *p* = 0.555. Perceived inclusion attainment became less positively related to all dimensions associated with agentic extraversion: It became unrelated to narcissistic admiration, *t*(307) = −0.17, *p* = 0.865, and remained positively but less strongly related to agency, *t*(307) = 3.63, *p* < 0.001, and boldness, *t*(307) = 2.03, *p* = 0.043. Perceived inclusion attainment became more negatively related to all dimensions associated with antagonism: narcissistic rivalry, *t*(307) = −4.45, *p* < 0.001, Machiavellian antagonism, *t*(307) = −6.07, *p* < 0.001, and meanness, *t*(307) = −6.60, *p* < 0.001. It remained positively related to planfulness, *t*(307) = 2.29, *p* = 0.023, and negatively related to disinhibition, *t*(307) = −4.51, *p* < 0.001.

The relations of the Dark Triad dimensions with assertiveness and affiliativeness were largely unchanged. Assertiveness remained positively associated with all dimensions associated with agentic extraversion: narcissistic admiration, *t*(306) = 10.08, *p* < 0.001, agency, *t*(306) = 17.67, *p* < 0.001, and boldness, *t*(306) = 16.57, β = 0.68, *p* < 0.001. It remained unrelated to narcissistic rivalry, *t*(306) = 0.42, *p* = 0.672, and meanness, *t*(306) = 1.15, *p* = 0.252, and positively related to Machiavellian antagonism, *t*(306) = 3.27, *p* < 0.001. It remained unrelated to planfulness, *t*(306) = 0.87, β = 0.05, *p* = 0.385, and disinhibition, *t*(306) = −1.13, β = −0.06, *p* = 0.262. Affiliativeness remained unrelated, or modestly positively related, to dimensions associated with agentic extraversion: narcissistic admiration, *t*(306) = 1.50, *p* = 0.134, agency, *t*(306) = 5.00, *p* < 0.001, and boldness, *t*(306) = 2.57, β = 0.11, *p* = 0.011. Affiliativeness remained negatively related to dimensions associated with antagonism: narcissistic rivalry, *t*(306) = −8.88, β = −0.45, *p* < 0.001, Machiavellian antagonism, *t*(306) = −13.47, *p* < 0.001, and meanness, *t*(306) = −13.80, β = −0.62, *p* < 0.001. It remained positively related to planfulness, *t*(306) = 3.23, β = 0.18, *p* = 0.001, and negatively related to disinhibition, *t*(306) = −7.67, *p* < 0.001.

### 3.4. Discussion

Study 2 replicates and extends Study 1. Consistent with Study 1, individuals high in Dark Triad traits had higher status aspirations and higher perceived status attainment. This included Machiavellianism when assessed with a measure that distinguishes it clearly from psychopathy (consistent with the results for the Trimmed-Mach in Study 1). In contrast, when status aspirations and perceived status attainment were controlled, Dark Triad traits were not related to inclusion aspirations or perceived inclusion attainment. This pattern also emerged for interpersonal behavior: Dark Triad traits related positively to assertiveness but negatively to affiliativeness. Thus, individuals high in Dark Triad traits may focus on agency over communion. They may desire high status, feel they have attained it reasonably well, and behave relatively dominantly in interpersonal situations, whereas they may not particularly desire inclusion, may not feel they have attained it particularly well, and may behave relatively disagreeably in interpersonal situations.

Dimension-specific results reveal which aspects of Dark Triad traits support these tendencies. Dimensions associated with agentic extraversion (narcissistic admiration, agency, and boldness) related positively to status aspirations, perceived status attainment, and assertiveness. In most cases, controlling inclusion aspirations or perceived inclusion attainment only strengthened these relations. Dimensions associated with antagonism (narcissistic rivalry, Machiavellian antagonism, and meanness) related positively to status aspirations and perceived status attainment, once inclusion aspirations and perceived inclusion attainment were controlled. However, they did not relate to assertiveness, except for a modest relation with Machiavellian antagonism. Lastly, dimensions related to impulsivity (planfulness and disinhibition) were not related to status aspirations, perceived status attainment, or assertiveness, once inclusion aspirations, perceived inclusion attainment, or affiliativeness were controlled. Thus, the links among status aspirations, perceived status attainment, assertiveness, and the Dark Triad appear to be driven primarily by agentic extraversion and antagonism rather than impulsivity. Notably, however, those high in antagonistic features may desire status more strongly, and feel they have attained it relatively well, but generally do not report behaving particularly assertively.

The results were markedly different for inclusion. The dimensions of the Dark Triad associated with agentic extraversion were unrelated or negatively related to inclusion aspirations once status aspirations were controlled; they were unrelated or modestly positively related (in the case of boldness) to perceived inclusion attainment once perceived status attainment was controlled; and they were unrelated or modestly positively related to affiliativeness once assertiveness was controlled. Individuals high on agentic extraversion dimensions may not be particularly interested in inclusion but may feel they are fairly well accepted by others and may behave in fairly affiliative ways. In contrast, dimensions associated with antagonism were unrelated or positively related to inclusion aspirations (in the case of narcissistic rivalry) but were consistently negatively related to perceptions of being included and to affiliativeness. Individuals high on these dimensions may not desire inclusion (aside from those high in narcissistic rivalry), may not feel they have attained it, and may not behave in particularly affiliative ways. Lastly, for dimensions associated with impulsivity, planfulness was unrelated to inclusion aspirations but positively related to perceived inclusion attainment and affiliativeness; disinhibition, in contrast, was positively related to inclusion aspirations but negatively related to inclusion attainment and affiliativeness. Thus, low impulsivity may facilitate gaining acceptance from others, whereas high impulsivity may undermine it.

## 4. General Discussion

The Dark Triad represents the socially aversive side of personality. Most efforts to understand the commonalities between these traits focus on shared structural personality correlates or common variance. In the present studies, we focused on examining the interpersonal motivations that characterize dark personalities to understand the potential functional bases of their behavioral tendencies (Jonason & Ferrell, 2016; Jonason & Zeigler-Hill, 2018). Extending research suggesting that narcissistic attributes function as a coordinated system to facilitate gaining status (Grapsas et al., 2020; Morf & Rhodewalt, 2001; Mahadevan, 2024; Zeigler-Hill et al., 2019), we found that individuals high in all Dark Triad traits are highly motivated to achieve status, in terms of being respected and admired by others. They also believe they have attained high status relatively well, once perceptions of inclusion attainment are controlled, and report behaving in ways likely to facilitate status attainment. These findings are consistent with the possibility that a common functional aspect of Dark Triad traits is their focus on the pursuit of social status.

In contrast, none of the Dark Triad traits were characterized by a desire for inclusion once desire for status was controlled. To the extent that individuals high in Dark Triad traits are motivated to be liked and accepted, it may reflect a general tendency to seek social validation, or to seek inclusion in order to attain status. Individuals high in Dark Triad traits also felt they were not particularly included (grandiose narcissism), or that they were relatively excluded (Machiavellianism and psychopathy), and reported not behaving in particularly affiliative ways. Our findings, overall, are consistent with prior evidence that those high in Dark Triad traits are high in agency but not communion (Jones & Paulhus, 2010). They are motivated to be respected and admired but not liked and accepted, behave in ways likely to help gain status but not inclusion, and perceive themselves to be high in status but not inclusion.

Our results complement earlier findings that individuals high in Dark Triad traits seek power, dominance, and hierarchical position (Jonason & Ferrell, 2016; Jonason & Zeigler-Hill, 2018; Lee & Ashton, 2005) by demonstrating that they are motivated to be admired and respected, specifically. Wanting to be admired and respected is a fundamental human motive (Anderson et al., 2015), which appears to be relatively pronounced in individuals high in Dark Triad traits. Taken together, those high in Dark Triad traits may more strongly desire status in terms of being respected and admired, reflecting prestige-based strategies for acquiring status, than inclusion in terms of being liked and accepted.

We also extend prior findings by controlling for desire for inclusion, highlighting the unique focus on status among those high in Dark Triad traits. Variance shared between desires for status and inclusion may reflect a drive for social validation; controlling this variance did not diminish the relation of Dark Triad traits to a desire for status but substantially weakened their relation to a desire for inclusion. Thus, those high in all Dark Triad traits appear to be relatively uniquely motivated to be respected and admired by others. Extending prior research, we found that all Dark Triad traits relate to perceptions of having relatively high status once perceptions of being included are controlled, and a tendency to behave assertively once affiliativeness is controlled.

Notably, we extend prior research by examining how the underlying dimensions of Dark Triad traits relate to the pursuit of both status and inclusion. There is increasing recognition that each Dark Triad trait is multidimensional and that their underlying dimensions can differentially predict important outcomes (Rose et al., 2023). With respect to the pursuit of status, dimensions related to conscientiousness or impulsivity seemed largely irrelevant. They may mainly facilitate (planfulness) or undermine (disinhibition) inclusion. Dimensions associated with agentic extraversion most strongly related to the desire for status, perceptions of having status, and assertiveness. Dimensions associated with antagonism also related positively to the desire for status and perceptions of having status but not assertiveness. These dimensions include dispositional tendences shared across all Dark Triad traits. They may reflect the core of a common drive to gain status.

### 4.1. Implications for Models of the Core of Dark Personalities

Our findings that Dark Triad traits share a drive for status are consistent with two accounts of the “core” of Dark Triad constructs, but our results for their underlying dimensions may have different implications for each perspective. The first is that Dark Triad traits reflect a fast life-history strategy (Jonason et al., 2010, 2013; McDonald et al., 2012). This perspective suggests Dark Triad traits “reflect a distinct system of solutions to adaptive problems that are characterized by limited mutualistic motives (e.g., kin protection) and enhanced individualistic motives (e.g., status seeking)” (Jonason & Zeigler-Hill, 2018, p. 132). If this perspective is correct, our results suggest that the Dark Triad features that constitute this system, with respect to pursuing status, may reflect agentic extraversion and antagonism but not impulsivity. Our findings suggest that agentic extraversion may be most instrumental in achieving status. Notably, though, we focus on prestige-related status. The antagonistic features of dark personalities may more strongly influence dominance-related strategies for pursuing status, such as intimidation and conflict (Zeigler-Hill et al., 2021).

The other perspective is that of the dark factor of personality, or D (Moshagen et al., 2018; Schreiber & Marcus, 2020). This perspective suggests that many dark personality features, including the Dark Triad, arise from a broad dispositional “tendency to maximize one’s individual utility—disregarding, accepting, or malevolently provoking disutility for others” (p. 657). In this perspective, status is one form of individual utility that a person high in dark personality features may pursue. A focus on D implies that personality features unique to specific “dark” personalities, such as planfulness in the case of Machiavellianism, and disinhibition in the case of psychopathy, are largely unrelated to D (Moshagen et al., 2020). Our results partly support this view. Planfulness and disinhibition—features unique to Machiavellianism and psychopathy—seemed largely irrelevant to status pursuit. On the other hand, agentic extraversion is not shared across all dark personalities considered to be manifestations of D (such as spitefulness, sadism, or greed). Thus, our finding that the Dark Triad dimensions related to agentic extraversion are related to the pursuit of status is somewhat inconsistent with D. Our results suggest that more than variance associated with D contributes to the shared character of those high in each Dark Triad trait, including their focus on the pursuit of status.

### 4.2. Limitations

We cannot establish causality given the correlational nature of our data. It is possible that Dark Triad traits lead people to desire status or that individuals who strongly desire status develop Dark Triad traits. Some of these links may be bi-directional: Dark Triad traits may push people to pursue and, in some cases, attain high status, which may then encourage their “dark” dispositional tendencies. Longitudinal or experimental research could more clearly establish causal directions across different time scales.

Our findings are also limited by using self-reports, which are susceptible to social desirability, shared method variance, and demand characteristics (Podsakoff et al., 2003). Some participants high in Dark Triad traits may be unwilling or unable to accurately report their “dark” dispositional tendencies (Carlson, 2013). Similarly, we assessed perceptions of status and inclusion attainment and retrospective reports of assertive and affiliative behavior and cannot make confident conclusions about actual social positions or behavior. Future research should supplement self-report with other methods of assessing personality and social outcomes, such as informant reports (Vazire & Mehl, 2008). There is reason, however, to believe that our findings are relevant to actual social positions. Perceptions of status and inclusion attainment are fairly accurate, correlating positively with informant reports (Anderson et al., 2006; Fournier, 2009).

Another limitation is that our samples consist of only U.S.A. residents recruited through Prolific.ac^TM^. Their age, ethnic, and gender identifications suggest a fairly diverse sample of the U.S.A. population, but these samples nevertheless reflect a narrow section of the diversity of human characteristics and experiences (Henrich et al., 2010). Future research should seek to examine the generalizability of these findings in other cultural contexts.

## 5. Conclusions

Individual differences in the Dark Triad traits of grandiose narcissism, Machiavellianism, and psychopathy may reflect individual differences in shared motivational patterns. We found that individuals high in all Dark Triad traits share a relatively strong desire for status, in the sense of being respected and admired, but no particularly strong desire for inclusion (once desire for status is controlled). These motivational predispositions relate most clearly to dimensions of the Dark Triad traits associated with agentic extraversion and antagonism but not impulsivity. Individuals high in Dark Triad traits felt they had achieved relatively high status, but not inclusion, and reported behaving in relatively assertive, but not affiliative, ways. Part of the “darkness” of these traits may thus be a shared motive to advance in the status hierarchy, by gaining respect and admiration, without a strong concern for being liked or accepted.

## Figures and Tables

**Table 1:** Study 1: Descriptive Statistics and Inter-Correlations for the Main Variables.

Variable	1	2	3	4	5	6	7
1. Status aspirations	1	-	-	-	-	-	-
2. Inclusion aspirations	0.59 ***	1	-	-	-	-	-
3. Perceived status attainment	0.34 ***	0.19 ***	1	-	-	-	-
4. Perceived inclusion attainment	0.17 **	0.16 **	0.73 ***	1	-	-	-
5. Grandiose narcissism	0.51 ***	0.31 ***	0.44 ***	0.26 ***	1	-	-
6. Machiavellianism	0.19 ***	0.11 ^†^	−0.18 **	−0.30 ***	0.20 ***	1	-
7. Psychopathy	0.30 ***	0.19 ***	0.09	−0.13 *	0.53 ***	0.48 ***	1
Mean	3.37	3.17	3.46	3.81	0.33	2.79	2.17
SD	0.81	0.89	0.86	0.74	0.21	0.44	0.66
Cronbach’s alpha	0.88	0.91	0.92	0.92	0.91	0.70	0.92

^†^ *p* < 0.10, * *p* < 0.05, ** *p* < 0.01, *** *p* < 0.001.

**Table 2:** Study 1 and 2: Standardized Regression Coefficients for Regression of Status- and Inclusion-Related Variables on the Dark Triad.

	Status Aspirations	Status Attainment	Inclusion Aspirations	Inclusion Attainment	Assertiveness	Affiliativeness
Grandiose narcissism (S1; NPI)	0.51 ***	0.53 ***	0.01	−0.12	-	-
Grandiose narcissism (S2; NARQ)	0.50 ***	0.58 ***	0.09	−0.19 ***	0.36 ***	−0.19 ***
Admiration	0.56 ***	0.65 ***	−0.01	−0.01	0.50 ***	0.07
Rivalry	0.20 ***	0.21 ***	0.18 ***	−0.34 ***	0.02	−0.45 ***
Machiavellianism (S1; MACH-IV)	0.20 ***	0.07	−0.01	−0.35 ***	-	-
Machiavellianism (S2; FFMI)	0.57 ***	0.61 ***	−0.35 ***	−0.19 ***	0.65 ***	−0.10 *
Agency	0.51 ***	0.54 ***	−0.34 ***	0.21 ***	0.70 ***	0.20 ***
Antagonism	0.30 ***	0.33 ***	−0.10	−0.46 ***	0.15 ***	−0.60 ***
Planfulness	0.02	−0.01	−0.08	0.18 ***	0.05	0.18 ***
Psychopathy (S1; SRP-III)	0.29 ***	0.39 ***	0.02	−0.41 ***	-	-
Psychopathy (S2; TPM)	0.33 ***	0.47 ***	−0.08	−0.32 ***	0.38 ***	−0.44 ***
Boldness	0.36 ***	0.51 ***	−0.24 ***	0.13 *	0.68 ***	0.11 *
Meanness	0.18 ***	0.35 ***	−0.01	−0.49 ***	0.05	−0.62 ***
Disinhibition	0.10	0.05	0.15 *	−0.34 ***	−0.06	−0.40 ***

* *p* < 0.05, *** *p* < 0.001.

**Table 3:** Study 2: Descriptive Statistics and Inter-Correlations for the Main Variables.

Variable	1	2	3	4	5	6	7	8
1. Status aspirations	1	−	−	−	−	−	−	−
2. Inclusion aspirations	0.55 ***	1	−	−	−	−	−	−
3. Perceived status attainment	0.39 ***	0.16 **	1	−	−	−	−	−
4. Perceived inclusion attainment	0.16 **	0.04	0.70 ***	1	−	−	−	−
5. Assertiveness	0.21 ***	−0.13 *	0.52 ***	0.45 ***	1	−	−	−
6. Affiliativeness	−0.04	0.02	0.25 ***	0.39 ***	0.01	1	−	−
7. Grandiose narcissism	0.55 ***	0.37 ***	0.44 ***	0.21 ***	0.36 ***	−0.19 ***	1	−
8. Machiavellianism	0.38 ***	−0.03	0.59 ***	0.40 ***	0.65 ***	−0.10 ^†^	0.54 ***	1
9. Psychopathy	0.29 ***	0.11 ^†^	0.25 ***	0.01	0.38 ***	−0.44 ***	0.56 ***	0.42 ***
Mean	3.41	3.06	3.56	3.91	0.85	2.29	3.09	1.97
SD	0.83	0.87	0.85	0.68	1.47	1.11	0.41	0.29
Cronbach’s alpha^4^	0.87	0.91	0.92	0.91	0.9	0.86	0.86	0.86

^†^ *p* < 0.10, * *p* < 0.05, ** *p* < 0.01, *** *p* < 0.001.

**Table 4:** Study 2: Inter-Correlations Among the Status- and Inclusion-Related Variables and the Dark Triad Dimensions.

Variable	1	2	3	4	5	6	7	8	9	10	11	12	13
1. Status aspirations	1	−	−	−	−	−	−	−	−	−	−	−	−
2. Inclusion aspirations	0.55 ***	1	−	−	−	−	−	−	−	−	−	−	−
3. Perceived status attainment	0.39 ***	0.16 **	1	−	−	−	−	−	−	−	−	−	−
4. Perceived inclusion attainment	0.16 **	0.04	0.70 ***	1	−	−	−	−	−	−	−	−	−
5. Assertiveness	0.21 ***	−0.13 *	0.52 ***	0.45 ***	1	−	−	−	−	−	−	−	−
6. Affiliativeness	−0.04	0.02	0.25 ***	0.39 ***	0.01	1	−	−	−	−	−	−	−
7. NARQ-Admiration	0.55 ***	0.30 ***	0.65 ***	0.44 ***	0.50 ***	0.08	1	−	−	−	−	−	−
8. NARQ-Rivalry	0.30 ***	0.30 ***	−0.03	−0.19 ***	0.02	−0.45 ***	0.27 ***	1	−	−	−	−	−
9. FFMI-Agency	0.32 ***	−0.06	0.68 ***	0.58 ***	0.70 ***	0.20 ***	0.64 ***	−0.11 *	1	−	−	−	−
10. FFMI-Antagonism	0.25 ***	0.07	0.02	−0.22 ***	0.14 **	−0.60 ***	0.22 ***	0.61 ***	−0.02	1	−	−	−
11. FFMI-Planfulness	−0.02	−0.07	0.12 *	0.18 **	0.05	0.18 **	0.04	−0.23 ***	0.23 ***	−0.24 ***	1	−	−
12. TPM-Boldness	0.23 ***	−0.04	0.60 ***	0.48 ***	0.68 ***	0.11 *	0.58 ***	0.04	0.79 ***	0.14 *	−0.02	1	−
13. TPM-Meanness	0.18 **	0.09 ^†^	0.01	−0.25 ***	0.05	−0.62 ***	0.12 *	0.62 ***	−0.08	0.78 ***	−0.34 ***	0.09	1
14. TPM-Disinhibition	0.18 **	0.20 ***	−0.19 ***	−0.31 ***	−0.06	−0.40 ***	0.03	0.44 ***	−0.34 ***	0.42 ***	−0.52 ***	−0.11 ^†^	0.52 ***

^†^ *p* < 0.10, * *p* < 0.05, ** *p* < 0.01, *** *p* < 0.001.

## Data Availability

The data and analysis codes are available at https://osf.io/79rfc/?view_only=c877b835480d4301a44f08f37f0e8ab6 (deposited on 1 April 2025).

## Notes

1 We additionally examined associations with Machiavellianism using the Trimmed-Mach, a 5-item version of the Mach-IV Scale (Rauthmann, 2013). The Mach-IV Scale may predominantly assess cynicism/misanthropy (Rauthmann & Will, 2011); the Trimmed-Mach was developed using the five scale items that best assess cynicism/misanthropy. The Trimmed-Mach has good psychometric properties. Its internal consistency, construct validity, and criterion validity equal or exceed those of the Mach-IV Scale (Rauthmann, 2013).

2 Status aspirations and inclusion aspirations both correlated positively with the Trimmed-Mach, *r*(279) = 0.32, *p* > 0.001, and *r*(279) = 0.13, *p* = 0.025, respectively. Perceived status attainment was uncorrelated with the Trimmed-Mach, *r*(279) = −0.01, *p* = 0.849, and perceived inclusion attainment correlated negatively with it, *r*(279) = −0.21, *p* < 0.001.

3 After controlling inclusion aspirations, status aspirations related positively to the Trimmed-Mach, *t*(278) = 5.18, β = 0.36, *p* < 0.001, whereas after controlling status aspirations, inclusion aspirations no longer related positively to the Trimmed-Mach, *t*(278) = −1.16, β = −0.08, *p* = 0.248. After controlling perceived inclusion attainment, perceived status attainment related positively to the Trimmed-Mach, *t*(278) = 3.59, β = 0.30, *p* < 0.001, whereas after controlling perceived status attainment, perceived inclusion attainment related negatively to the Trimmed-Mach, *t*(278) = −5.15, β = −0.43, *p* < 0.001. Thus, when Machiavellianism was assessed via the Trimmed-Mach, it, like grandiose narcissism and psychopathy, related positively to status aspirations and perceived status attainment (after controlling inclusion aspirations and perceived inclusion attainment, respectively), but not to inclusion aspirations and perceived inclusion attainment (after controlling status aspirations and perceived status attainment, respectively).

4 All Dark Triad sub-scales showed good internal consistency: NARQ-Admiration (*M* = 2.83, *SD* = 0.83, α = 0.90), NARQ-Rivalry (*M* = 2.18, *SD* = 0.90, α = 0.85), FFMI-Agency (*M* = 3.39, *SD* = 0.73, α = 0.92), FFMI-Antagonism (*M* = 2.46, *SD* = 0.55, α = 0.83), FFMI-Planfulness (*M* = 3.77, *SD* = 0.67, α = 0.73), TPM-Boldness (*M* =2.49, *SD* = 0.45, α = 0.79), TPM-Meanness (*M* = 1.58, *SD* = 0.43, α = 0.88), and TPM-Disinhibition (*M* = 1.78, *SD* = 0.45, α = 0.85).
